# STING-dependent trained immunity contributes to host defense against *Clostridium perfringens* infection via mTOR signaling

**DOI:** 10.1186/s13567-024-01301-1

**Published:** 2024-04-15

**Authors:** Zhen-Zhen Liu, Cheng-Kai Zhou, Xiao-Qi Lin, Yu Gao, Xue-Yue Luo, Jia-Bao Zhang, Qi Yin, Liang Zhang, Jian-Gang Zhang, Xin An, Wei Chen, Yong-Jun Yang

**Affiliations:** State Key Laboratory for Diagnosis and Treatment of Severe Zoonotic Infectious Diseases, Key Laboratory for Zoonosis Research of the Ministry of Education, Institute of Zoonosis, and College of Veterinary Medicine, Jilin University, Changchun, 130062 China

**Keywords:** STING, macrophages, innate immune, trained immunity, *Clostridium perfringens*

## Abstract

*Clostridium perfringens* (*C. perfringens*) infection is recognized as one of the most challenging issues threatening food safety and perplexing agricultural development. To date, the molecular mechanisms of the interactions between *C. perfringens* and the host remain poorly understood. Here, we show that stimulator of interferon genes (STING)-dependent trained immunity protected against *C. perfringens* infection through mTOR signaling. Heat-killed *Candida albicans* (HKCA) training elicited elevated TNF-α and IL-6 production after LPS restimulation in mouse peritoneal macrophages (PM). Although HKCA-trained PM produced decreased levels of TNF-α and IL-6, the importance of trained immunity was demonstrated by the fact that HKCA training resulted in enhanced bacterial phagocytic ability and clearance in vivo and in vitro during *C. perfringens* infection. Interestingly, HKCA training resulted in the activation of STING signaling. We further demonstrate that STING agonist DMXAA is a strong inducer of trained immunity and conferred host resistance to *C. perfringens* infection in PM. Importantly, corresponding to higher bacterial burden, reduction in cytokine secretion, phagocytosis, and bacterial killing were shown in the absence of STING after HKCA training. Meanwhile, the high expression levels of AKT/mTOR/HIF1α were indeed accompanied by an activated STING signaling under HKCA or DMXAA training. Moreover, inhibiting mTOR signaling with rapamycin dampened the trained response to LPS and *C. perfringens* challenge in wild-type (WT) PM after HKCA training*.* Furthermore, STING‑deficient PM presented decreased levels of mTOR signaling-related proteins. Altogether, these results support STING involvement in trained immunity which protects against *C. perfringens* infection via mTOR signaling.

**Supplementary Information:**

The online version contains supplementary material available at 10.1186/s13567-024-01301-1.

## Introduction

*Clostridium perfringens* (*C. perfringens*) is an opportunistic pathogen of humans and livestock, causing a range of serious enteric and histotoxic infections, including gas gangrene, enteritis/enterocolitis, and enterotoxemia [1]. In addition, *C. perfringens* can be present as a contaminant in meat, vegetables, and raw milk products. Therefore, it is frequently associated with foodborne outbreaks and classified as the second most prevalent etiological factor of bacterial food poisoning in the United States, Europe, and many other regions [2]. Traditional toxoid vaccines and antibiotics are effective countermeasures in combating *C. perfringens* infection. With the drug resistance spectrum expanding and waning vaccine immunity, the demand for novel control strategies is even more pressing.

The innate immune response serves as the initial barrier of host defense against pathogenic infections. Research on the anti-bacterial innate immune response is beneficial for finding the intervention target. According to recent studies, the innate immune cells (such as monocytes, macrophages, NK cells, and neutrophils) can achieve memory characteristics after primary stimulation and mount host response to restimulation, a phenomenon known as trained immunity or innate immune memory, accompanied by the epigenetic and metabolic reprogramming [3]. In contrast to adaptive immunity, trained immunity is nonspecific, rapid, modest, and exists for a relatively short time. One notable feature of trained immunity is the occurrence of the Warburg effect, wherein cells undergo a metabolic transition from oxidative phosphorylation to aerobic glycolysis. The PI3K/Akt/mTOR pathway and the TCA cycle appear to be the common denominators in this process, and their interplay influences the regulation of histone acetylation and methylation in the promoters and enhancers of genes responsible for encoding inflammatory cytokines [1]. Many studies have reported evidence for trained immunity in plants, invertebrates, and vertebrates [4]. Trained immunity can be activated by diverse stimuli, such as β-glucan, LPS, LTA, BCG, a Western diet, and oxLDL [1, 5–11]. It is like a double‐edged sword. The induction of trained immunity could both promote increased susceptibility to secondary infections and contribute to the progression of the inflammatory disorder [12]. Precisely due to the memory property, trained immunity provides protection against subsequent heterologous pathogenic infections, including *Mycobacterium tuberculosis*, *Candida albicans*, *Leishmania braziliensis*, and *influenza A virus* [13–15]. Further elucidation in the field of trained immunity is likely to open new avenues for the novel preventive and therapeutic strategies of host resistance to *C. perfringens* infection.

Germline-encoded pattern-recognition receptors (PRR) act as “sensors” of trained immunity. At present, only a proportion of the NOD-like receptors, the Toll-like receptors, and the C-type lectin receptors have been characterized for their roles in trained immunity [6, 16–18]. Nevertheless, the function of nucleic acid receptors and related signaling proteins has not yet been determined during the production of trained immunity. The adaptor protein stimulator of interferon genes (STING) has been widely investigated for its role in DNA sensing. Its function primarily revolves around the activation of type I interferons (IFN), which are mainly involved in the development of infectious diseases, autoimmune diseases, and cancer [19]. Its role in regulating trained immunity and the impact of STING-mediated trained immunity on antibacterial infection remains elusive. Herein, we demonstrate that STING-dependent trained immunity contributes to host defense against *C. perfringens* infection via mTOR signaling.

## Materials and methods

### Mice

C57BL/6 J wild-type (WT) mice and STING^−/−^ mice were procured from Jackson Laboratory. The mice were provided with sterilized food and water, and were subjected to a strict 12 h light cycle. They were housed in groups of up to 6 mice per cage. Age-and sex-matched mice were used for all experiments. All animal studies were conducted in accordance with the approved experimental practices and standards set by the Animal Welfare and Research Ethics Committee at Jilin University (KT202202182).

### Trained immunity in vitro model

Peritoneal macrophages (PM) were obtained from WT mice at 3 days after mice were injected with 4% thioglycollate broth (Sigma-Aldrich, #70157). The PM were distributed in 96-well cell culture plates, each well containing 2 × 10^5^ cells, or in 6-well cell culture plates, each well containing 3 × 10^6^ cells. Subsequently, the PM were stimulated using RPMI1640 medium (Gibco, #31800–022), DMXAA (50 ug/mL, Sigma-Aldrich, #D5817), or heat-killed *Candida albicans* (HKCA, 1 × 10^5^ cells/mL) for 24 h. Then, cells were washed and rested for 5 days in the culture medium with 10% FBS (Gibco, #A31608-02). On day 6, a final wash was conducted, followed by stimulation with medium, 100 ng/mL of LPS (Sigma-Aldrich, #L6529), and *C. perfringens* (ATCC13124, MOI = 5). The cell supernatants and lysates were subsequently collected to perform ELISA or Western blotting assays.

### Trained immunity in vivo model

Age- and sex-matched WT and STING^−/−^ mice were subjected to training with two intraperitoneal (i.p.) injections of 1 × 10^6^ cells of HKCA on days −7 and −4. Phosphate-buffered saline (PBS) was used as the control. For in vitro experiments, mice were injected i.p. with 4% thioglycolate broth on day −3. PM were collected and stimulated on day 0 and day 1, respectively. For in vivo experiments, mice were subjected to intramuscular infection with a concentration of 2 × 10^7^ colony-forming units (CFU) of *C. perfringens* diluted in PBS in a total volume of 100 μL on day 0. After 24 h post-infection, the mice were euthanized to obtain infected leg muscle samples for the purpose of quantifying bacterial load. Additionally, the severity of gas gangrene was evaluated using a scoring system that had been previously described in a study [20].

### Phagocytosis and intracellular killing assays

PM were infected with *C. perfringens* at a multiplicity of infection (MOI: 5). Following infection, the PM were centrifuged at 515 × *g* for 2 min to ensure synchronous infection. Subsequently, the cells were incubated at 37 °C for 1 h and washed twice with RPMI-1640 medium. The PM were then cultured in RPMI-1640 medium supplemented with 200 U/mL penicillin, 200 U/mL streptomycin, and 100 μg/mL gentamicin to eliminate excessive extracellular bacteria for 1 h. Finally, the PM were incubated at 37 ℃ for the specified time intervals. Killing efficiency was calculated as ([CFU/mL at 2 h] − [CFU/mL at 4 h])/(CFU/mL at 2 h).

### Real‑time PCR

RNA was extracted using TRI reagent (Sigma-Aldrich, #T9424) and converted into cDNA. Subsequently, real-time PCR assays were conducted using SYBR Green (Roche, #4913914001) on an ABI Prism 7500 sequence detection system (Life Tech [Applied BioSystems], Waltham, USA). The Agilent integrity check was used to verify RNA quality. Gene expression levels were determined using the 2^−ΔCt^ method. The following primer sequences were utilized: β-actin sense 5′-CGTGGGCCGCCCTAGGCACCA-3′ and antisense 5′-TTGGCCTTAGGGTTCAGGGGGG-3′. IFN-β sense 5′-ACTGCCTTTGCCATCCAAGA-3’ and antisense 5’-CACTGTCTGCTGGTGGAGTT-3’. CXCL10 sense 5′-ATCCCTGCGAGCCTATC-3’ and antisense 5′-GCCATCCACTGGGTAAA-3′.

### Cytokine measurements

The supernatants were used for ELISA measurements according to the manufacturer’s instructions. Mouse TNF-α and IL-6 ELISA kits were purchased from R&D Systems (#DY410 and #DY406).

### Western blotting analysis

PM were harvested and lysed using cold RIPA lysis buffer supplemented with complete protease inhibitor cocktail (Sigma-Aldrich, #P8340). Samples were centrifuged and supernatants were used for immunoblotting. The cell lysates were separated by SDS-PAGE and transferred to PVDF membranes (Millipore, # ISEQ00010). Following blocking with 5% nonfat milk, the membranes were blotted with antibodies against p-mTOR (Abcam, #ab109268), p-AKT (Proteintech, #66444-1-Ig), HIF1α (Abcam, #ab179483), STING (Proteintech, #19851-1-AP), p-TBK1 (Cell Signaling Technology, #5483), p-IRF3 (Cell Signaling Technology, #29047), and β-actin (Proteintech, #81115-1-RR).

### Statistical analysis

The experiments were conducted independently on three occasions. The differences between mean values were assessed with Student’s *t*-test, one-way ANOVA with Dunnett multiple comparison test, or two-way ANOVA with Bonferroni multiple comparison test (*P < 0.05; **P < 0.01; ***P < 0.001). Data are shown as mean ± SEM. The statistical significance was determined by the software GraphPad Prism6.

## Results

### HKCA-induced trained immunity enhances macrophage function and promotes host defense against *C. perfringens* infection

**Figure 1:**
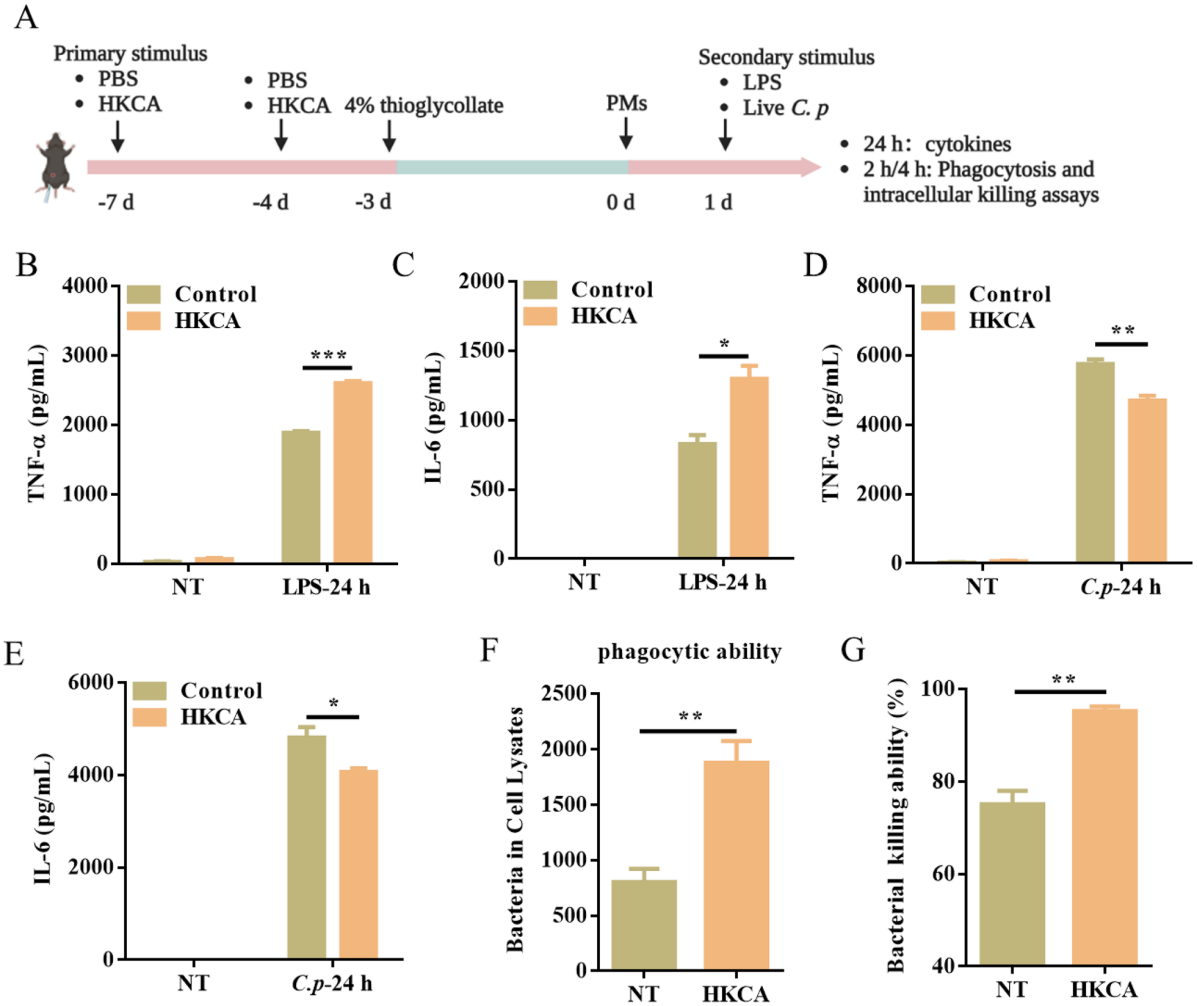
**Trained immunity protects the host against C. perfringens infection.** **A** Flow chart of in vitro trained immunity experimental procedure. (**B**−**E**) TNF-α and IL-6 production was measured in the supernatants of mouse PM response to LPS or *C. perfringens* stimulation according to A (*n* = 3 independent experiments). **F**, **G** Phagocytosis and intracellular killing of *C. perfringens* was detected by CFU enumeration. Data are shown as mean ± SEM. Data were pooled from 3 independent experiments. Statistical significance is indicated by *p < 0.05, **p < 0.01, and ***p < 0.001.

To evaluate the role of trained immunity in host defense against *C. perfringens* infection, an in vitro model was established for trained immunity. WT mice were trained with two i.p. injections of HKCA on days -7 and -4. PBS was used as the control. On day -3, mice were injected i.p. with 4% thioglycolate broth to induce macrophage differentiation and accumulation. Then PM were collected and restimulated (LPS or *C. perfringens*) on day 0 and day 1, respectively (Figure 1A). We found that HKCA training induced a significant increase in the concentrations of TNF-α and IL-6 upon LPS stimulation (Figure 1B, C). Unlike LPS, there was a lower production of TNF-α and IL-6 response to *C. perfringens* infection under HKCA training compared with the control group (Figure 1D, E). Based on the aforementioned results, we further analyzed the phagocytosis and bacterial killing ability of PM before and after HKCA training. Interestingly, HKCA training resulted in enhanced bacterial phagocytic ability and clearance (Figure 1F, G). Collectively, these results suggest that HKCA-induced trained immunity enhances macrophage function and promotes host defense against *C. perfringens* infection.

### Trained immunity activates STING signaling

**Figure 2:**
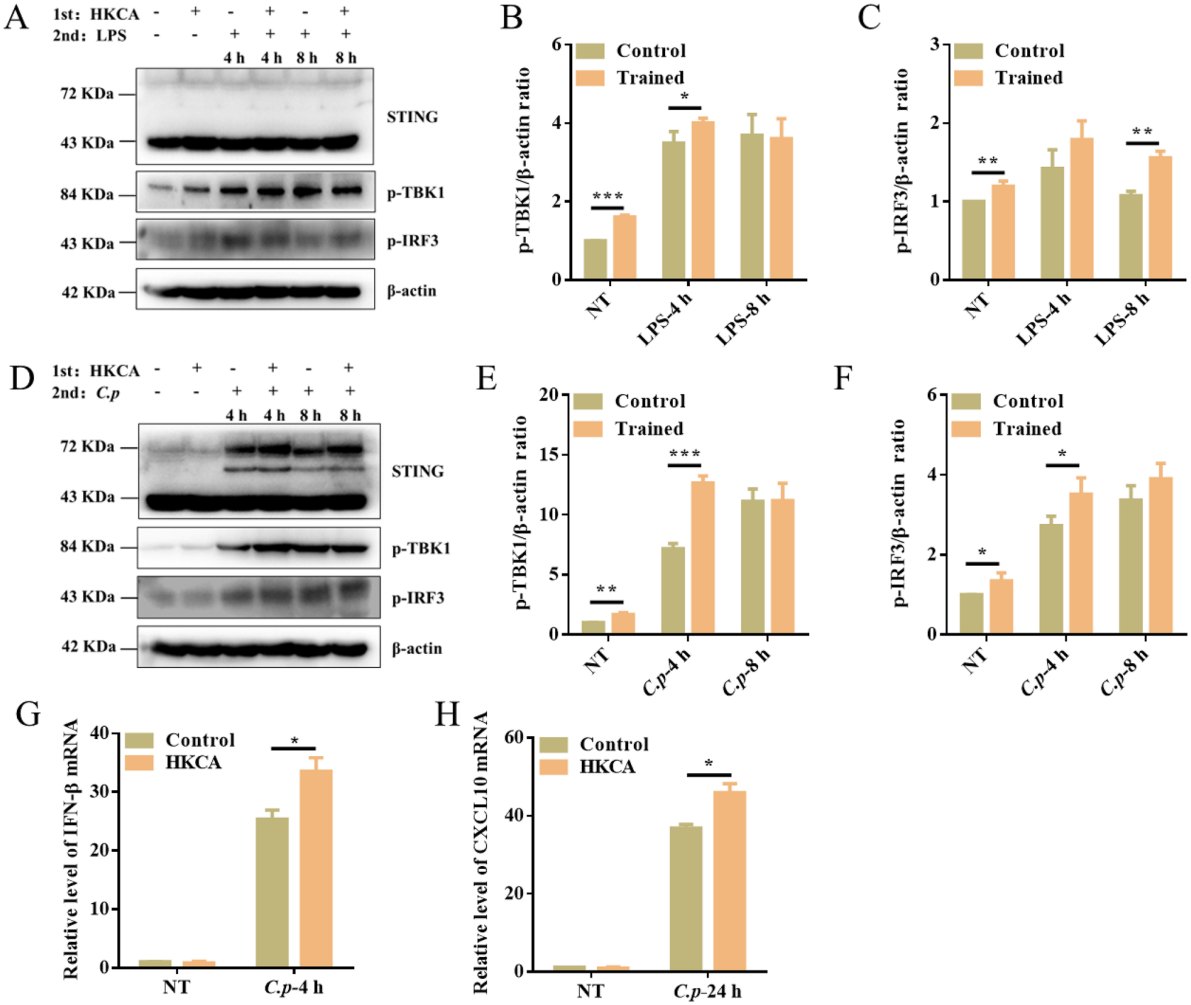
**Trained immunity activates STING signaling.** WT PM were trained with HKCA and then were restimulated with LPS or *C. perfringens* at an MOI of 5 for the indicated times. **A**, **D** Whole cell lysates were analyzed for STING, p-TBK1, p-IRF3, and β-actin by Western blotting. **B**, **C**, **E**, and **F** The gray intensity value of proteins was calculated using ImageJ software. **G** IFN-β mRNA levels were measured at 4 h post‑infection by qRT‑PCR. **H** CXCL10 mRNA levels were measured at 24 h post‑infection by qRT‑PCR. Data are shown as mean ± SEM. Data were pooled from 3 independent experiments. Statistical significance is indicated by *p < 0.05, **p < 0.01, and ***p < 0.001.

STING is recognized as a significant adaptor protein of DNA sensors. Its role in regulating trained immunity and the impact of STING-mediated trained immunity on antibacterial infection remains elusive. To investigate STING signaling activation during the process of trained immunity, we detected the protein levels of STING, p-TBK1, and p-IRF3 before and after stimulation with LPS or *C. perfringens*. The results show that STING signaling was upregulated in all HKCA-trained samples compared with their controls (Figure 2A–F). Meanwhile, the expression levels of CXCL10 and IFN-β were significantly increased in HKCA training PM compared with the control cells after *C. perfringens* infection (Figure 2G , H). These results suggest that trained immunity induces STING signaling activation.

### STING agonist DMXAA is a strong inducer of trained immunity and confers host resistance to *C. perfringens* infection in macrophages

**Figure 3:**
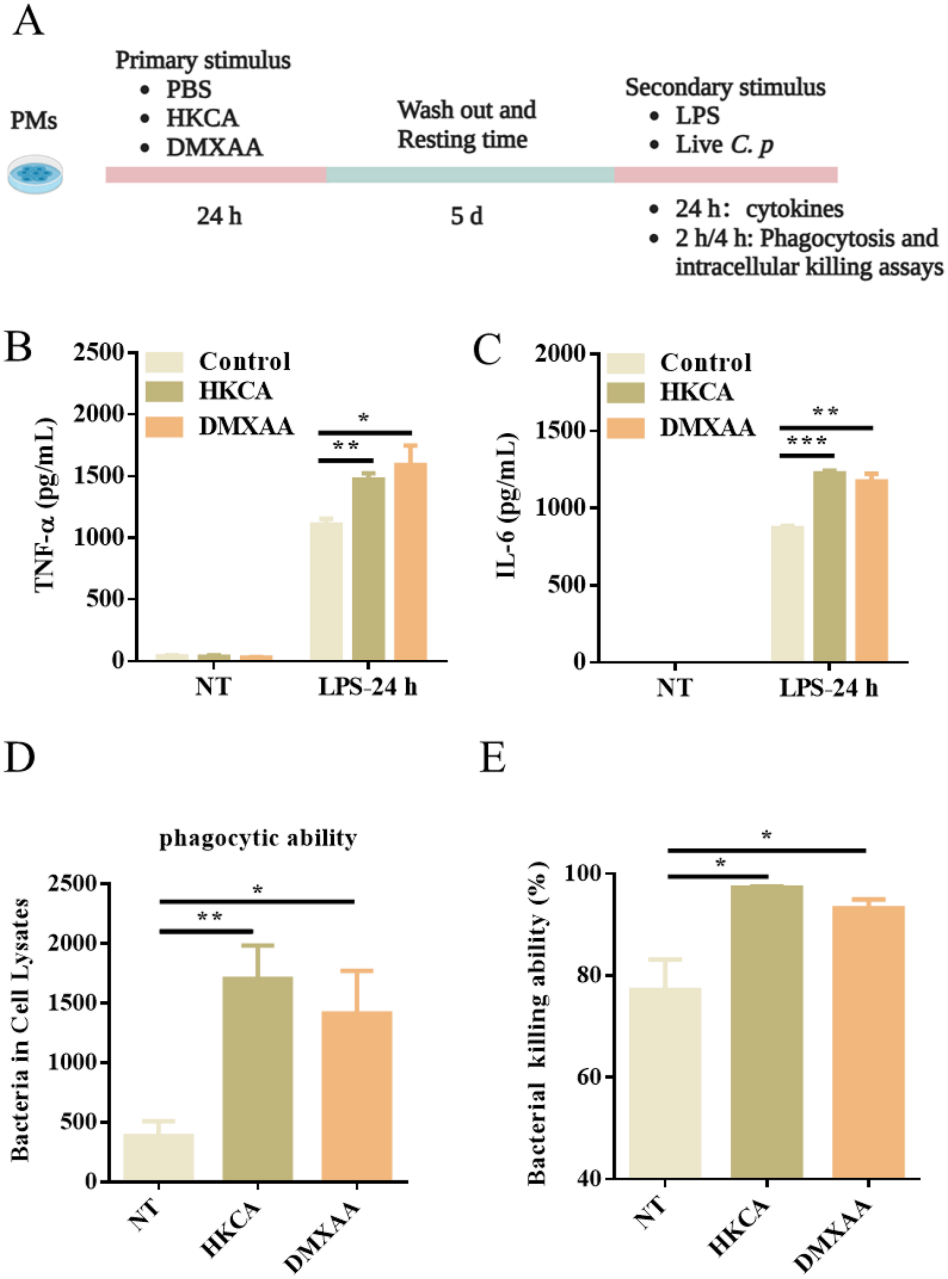
**STING agonist DMXAA directly induces trained immunity.** WT PM were trained with DMXAA or HKCA for 24 h and rested for 5 days. WT PM were restimulated with LPS or *C. perfringens* at an MOI of 5 for the indicated times. **A** Flow chart of in vitro trained immunity experimental procedure. **B**, **C** TNF-α and IL-6 production were measured in the supernatants of mouse PM response to LPS stimulation according to A. **D**, **E** Phagocytosis and intracellular killing of *C. perfringens* was detected by CFU enumeration. Data are shown as mean ± SEM. Data were pooled from 3 independent experiments. Statistical significance is indicated by *p < 0.05, **p < 0.01, and ***p < 0.001.

To further examine the potential involvement of STING in trained immunity, we trained PM with STING agonist DMXAA for 24 h and rested for 5 d to induce macrophage differentiation. HKCA and PBS served as positive and negative control, respectively. Subsequently, the cytokine production was measured after treatment with LPS (Figure 3A). We found that training with DMXAA showed a higher production of IL-6 and TNF-α upon LPS stimulation compared with the control group, nearly to the extent observed in the HKCA training group. (Figure 3B, C). We further characterized the phagocytosis and the bacterial killing ability of PM before and after DMXAA training. Expectedly, DMXAA training resulted in enhanced bacterial phagocytic ability and clearance (Figure 3D , E). These results indicate that DMXAA is a strong inducer of trained immunity and confers host resistance to *C. perfringens* infection in macrophages.

### STING deficiency impairs trained immunity and has a defect in bacterial clearance

**Figure 4:**
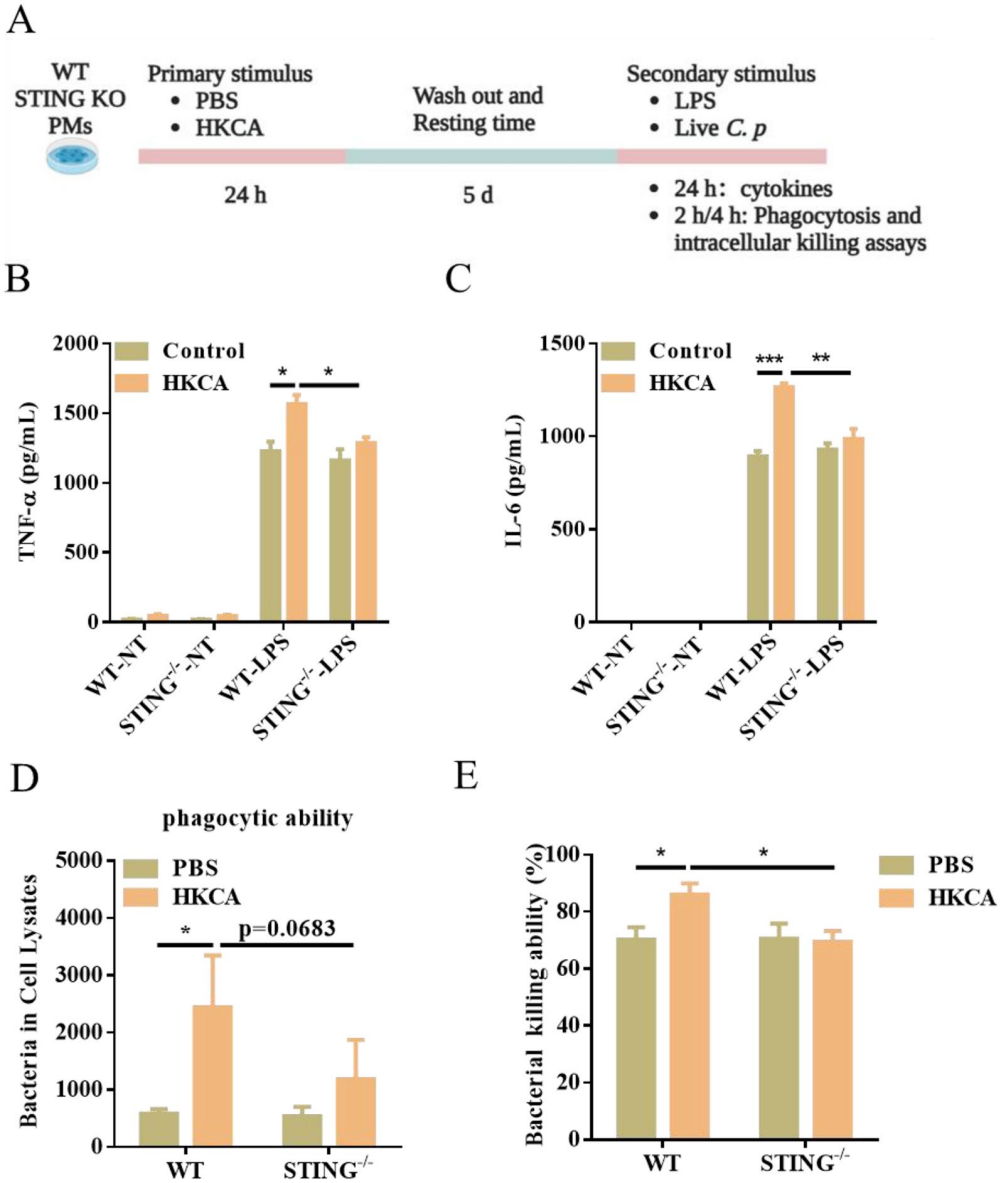
**STING deficiency dampens the trained response to LPS stimulation and C. perfringens infection.** WT and STING^−/−^ PM were trained with HKCA and then were restimulated with LPS or *C. perfringens* at an MOI of 5 for the indicated times. **A** Flow chart of in vitro trained immunity experimental procedure. **B**, **C** TNF-α and IL-6 production was measured in the supernatants of mouse PM response to LPS stimulation according to A. **D**, **E** Phagocytosis and intracellular killing of *C. perfringens* was detected by CFU enumeration. Data are shown as mean ± SEM. Data were pooled from 3 independent experiments. Statistical significance is indicated by *p < 0.05, **p < 0.01, and ***p < 0.001.

Given the strong relationship between STING and trained immunity, our study aimed to investigate whether STING deficiency impairs trained immunity following HKCA training. WT and STING^−/−^ mice were trained with two i.p. injections of HKCA, and then PM were collected and restimulated with LPS and *C. perfringens* (Figure 4A). As expected, we found that PM from WT mice training with HKCA exhibited a heightened production of IL-6 and TNF-α in response to LPS stimulation compared with the control group, whereas no significant differences were observed in STING^−/−^ PM in comparison with before and after HKCA training (Figure 4B , C). In accordance with the above results, HKCA training did not impact bacterial phagocytic ability and clearance in STING^−/−^ PM (Figure 4D, E). STING^−/−^ PM presented decreased levels of IL-6 and TNF-α compared to WT PM after infection with *C. perfringens*, stimulated or not with HKCA (Additional files 1A and B). Together, STING plays a crucial role in inducing protective trained immunity.

### STING is dependent on the Akt/mTOR/HIF1α pathway in HKCA-induced trained immunity

**Figure 5:**
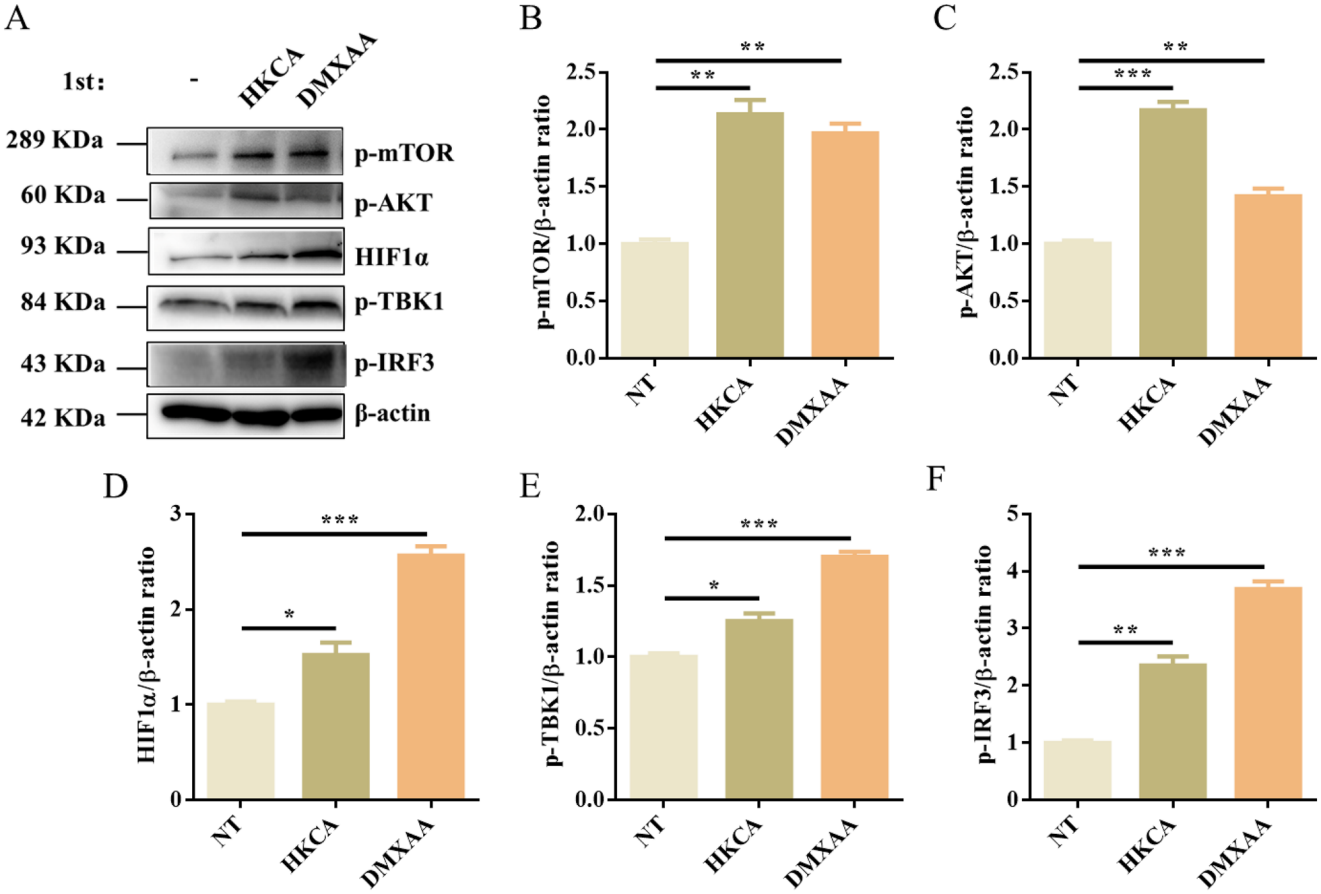
**DMXAA or HKCA training activates mTOR signaling.** WT PM were trained with DMXAA or HKCA for 24 h and rested for 5 days. **A** The cellular lysate was analyzed for p-mTOR, p-AKT, HIF1α, p-TBK1, p-IRF3, and β-actin by Western blotting. **B**–**F** The gray intensity value of proteins was calculated using ImageJ software. Data are shown as mean ± SEM. Data were pooled from 3 independent experiments. Statistical significance is indicated by *p < 0.05, **p < 0.01, and ***p < 0.001.

**Figure 6:**
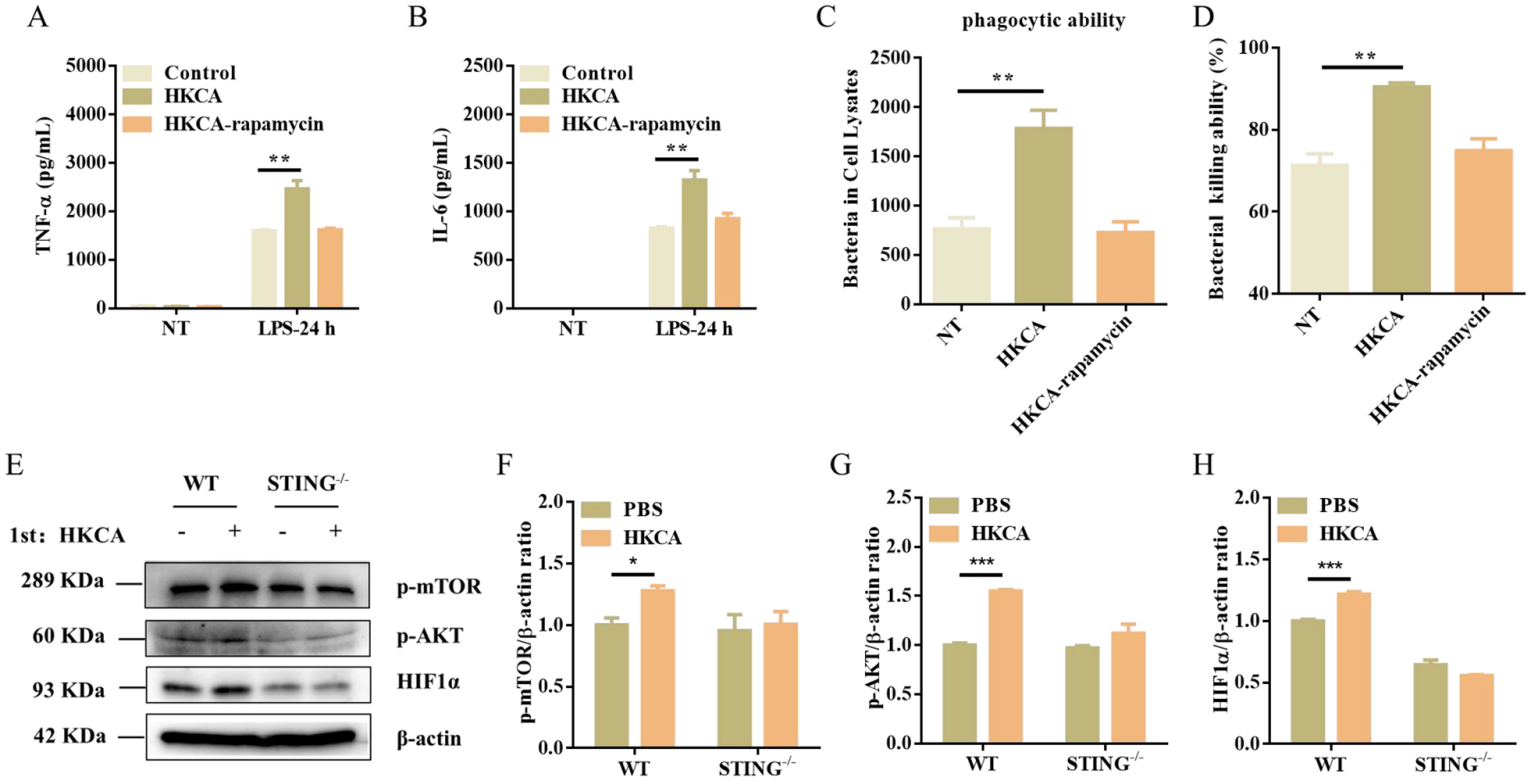
**Akt/mTOR/HIF1α pathway is indispensable for STING-mediated trained immunity.** PM were pretreated with rapamycin (10 μM) for 1 h, and then the cells were trained with HKCA for 24 h and rested for 5 days. PM were restimulated with LPS or *C. perfringens* at an MOI of 5 for the indicated times. **A**, **B** TNF-α and IL-6 production were measured in the supernatants of PM response to LPS stimulation. **C**, **D** Phagocytosis and intracellular killing of *C. perfringens* was detected by CFU enumeration. **E** The cellular lysate was analyzed for p-mTOR, p-AKT, HIF1α, and β-actin by Western blotting. **F**–**H** The gray intensity value of proteins was calculated using ImageJ software. Data are shown as mean ± SEM. Data were pooled from 3 independent experiments. Statistical significance is indicated by *p < 0.05, **p < 0.01, and ***p < 0.001.

These findings prompted us to further investigate the specific mechanism of the regulation of HKCA-induced trained immunity by STING. Since mTOR is a key regulator of β-glucan-induced trained immunity in monocytes, we sought to determine if mTOR signaling is also required for STING-mediated trained immunity in macrophages. Surprisingly, HKCA or DMXAA training both activated the mTOR and STING signaling pathways, as shown by the increased levels of p-AKT, p-mTOR, HIF1α, p-TBK1, and p-IRF3 compared to the control cells before secondary stimulation (Figure 5A–F). When mTOR inhibitor rapamycin was added, HKCA training-induced proinflammatory cytokine (TNF-α and IL-6) production was significantly decreased upon LPS stimulation compared with the control group (Figure 6A, B). Consistently, the addition of rapamycin reduced the bacterial phagocytic ability and clearance upon *C. perfringens* infection (Figure 6C, D). Consistent with the above results, we found that STING^−/−^ PM displayed notably reduced levels of p-AKT, p-mTOR, and HIF1α after HKCA training compared with those in WT PM before secondary stimulation (Figure 6E–H). Collectively, these results indicate that STING deficiency leads to a defect in trained immunity by impairing the mTOR signaling pathway.

### STING-dependent trained immunity contributes to host protection against *C. perfringens* soft tissue infection

**Figure 7:**
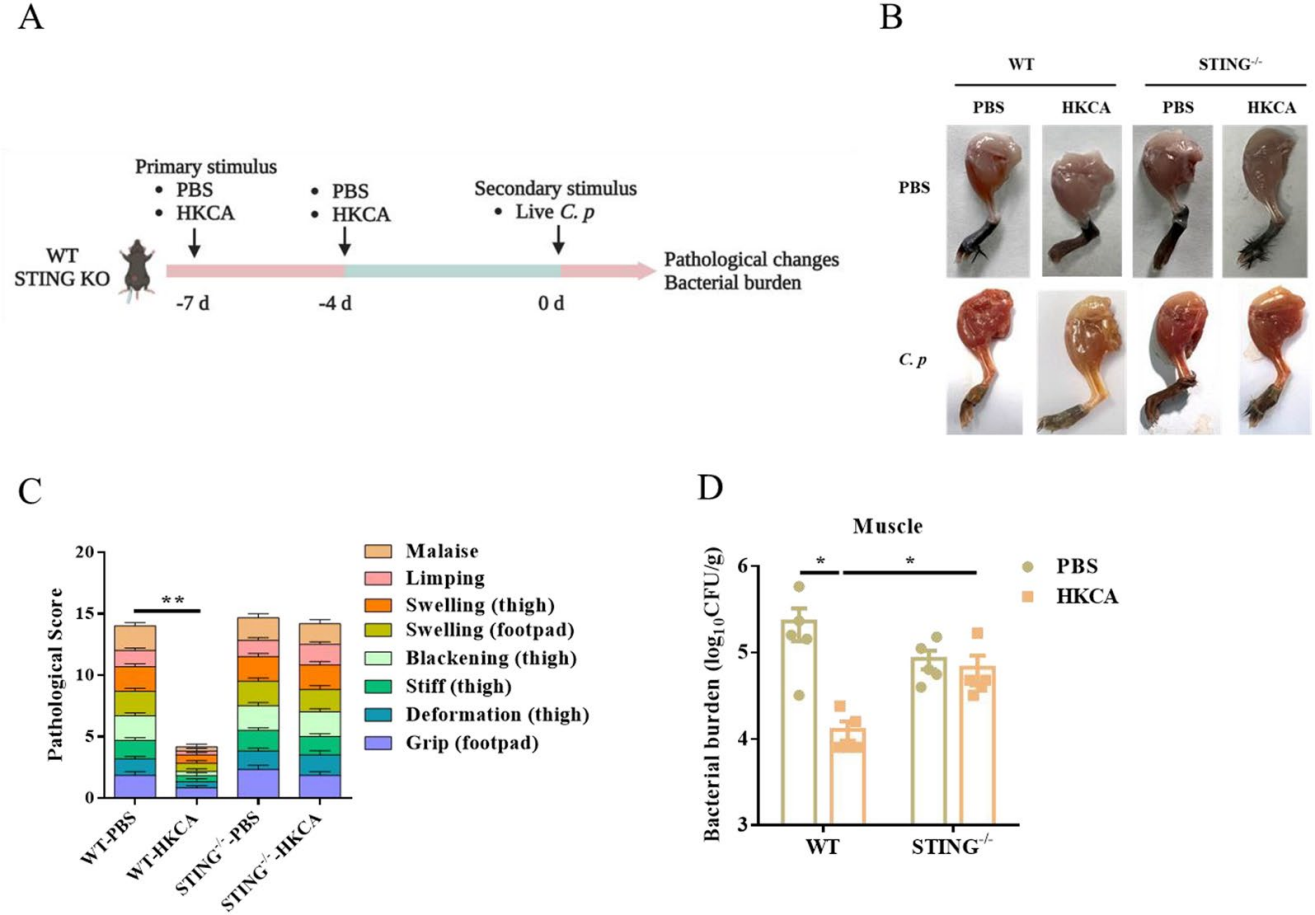
**STING-dependent trained immunity protects mice from C. perfringens soft tissue infection.** Age- and sex-matched WT and STING^−/−^ mice were trained with two i.p. injections of HKCA on days −7 and −4. PBS was used as the control. 7 days later, mice were intramuscularly infected with 2 × 10^7^ CFU of *C. perfringens* for 24 h. **A** Flow chart of in vivo trained immunity experimental procedure. **B** Representative gross images of legs. **C** Cumulative gross pathology scores. **D** Bacterial counts. Data are shown as mean ± SEM. Data were pooled from 3 independent experiments. Statistical significance is determined by *p < 0.05 and **p < 0.01.

We further established a gas gangrene model to validate the role of HKCA-induced trained immunity during *C. perfringens* infection in vivo. WT and STING^−/−^ mice were trained with two i.p. injections of HKCA on days -7 and -4. 7 days later, mice were intramuscularly infected with 1 × 10^7^ CFU of *C. perfringens* for 24 h (Figure 7A). Mice developed gas gangrene in the control group, characterized by marked swelling and hemorrhage. The HKCA training group exhibited much milder symptoms in WT mice, but there was no significant difference in STING^−/−^ mice before and after HKCA training (Figure 7B, C). In line with this, the HKCA training group harbored dramatically decreased loads of *C. perfringens* in the muscles of WT mice. No significant change in bacterial burden was observed in STING^−/−^ mice before and after HKCA training (Figure 7D). These findings suggest that STING-dependent trained immunity enhances host resistance to *C. perfringens* soft tissue infection.

## Discussion

*C. perfringens* is an opportunistic pathogen that ubiquitously spreads in the abiotic environment and biotic intestinal tracts. Due to its diverse virulence factors and strong resistance to adversity, *C. perfringens* causes a wide variety of diseases including intestinal or foodborne diseases as well as gangrenes, which harms both human health and the development of agriculture. A crucial first step in tackling this problem is the detailed understanding of the host's innate immune response against *C. perfringens* infection based on the relative paucity of research. At present, only a few studies have demonstrated that toll-like receptor 4 accelerates granulopoiesis and enhances host defense against *C. perfringens* infection [21]. The toxins lecithinase and perfringolysin O of *C. perfringens* have been found to activate the NLRP3 inflammasome, and NLRP3 deficiency leads to increased host susceptibility to *C. perfringens* infection [22, 23]. Consequently, the need for new preventive and therapeutic strategies has become more urgent.

Trained immunity offers interesting perspectives for dual memory vaccine designing and treatment strategies in infectious diseases. In our study, there is a novel finding that STING-dependent trained immunity protected against *C. perfringens* infection by regulating mTOR signaling. Using a trained immunity model in PM, we first demonstrate that HKCA training induced significant elevations of TNF-α and IL-6 after LPS restimulation. This was consistent with the “classic” trained immunity model in human monocytes/macrophages [24]. Notably, HKCA-trained PM exhibited decreased levels of cytokines but enhanced bacterial phagocytic ability and clearance response to *C. perfringens* infection. We speculate that this may be due to the intertwined interactions between inflammation and infection, both of which are mutually the cause or consequence. As has been reported previously, trained immunity confers broad-spectrum protection against bacterial infections, whereas the trend of cytokine secretion is inconsistent with each other [25]. These results demonstrate that HKCA-induced trained immunity enhances macrophage function and promotes host defense against *C. perfringens* infection.

As an important component of the innate immune system, STING functions as an adaptor molecule for intracellular DNA sensors (such as cGAS, IFI16, and DNA-PK) in addition to as a direct sensor of cyclic dinucleotides [26–29]. Traditionally, STING activation induces type I interferon responses which have been associated with inflammation, infection, cancer, and autoimmune diseases. However, the biological implications of STING in the process of trained immunity have not been characterized. In this study, we found that STING signaling was upregulated in all HKCA-trained samples before and after stimulation with LPS or *C. perfringens*. To further explore the underlying causes, we trained PM with the STING agonist DMXAA. Intriguingly, our results show that DMXAA was a strong inducer of trained immunity, characterized by increased responsiveness to LPS challenge and enhanced phagocytosis and bacterial killing ability to *C. perfringens* infection. Importantly, using STING^−/−^ mice, we further show that STING deficiency impairs trained immunity and has a defect in bacterial clearance. These results align with previous research demonstrating that a recombinant BCG expressing a STING agonist shows enhanced antitumor efficacy by triggering trained immunity remodeling [30]. Taken together, STING acts as a key regulator and maintainer of trained immunity.

Epigenetic and metabolic reprogramming underlies the induction of trained immunity. mTOR functions as a master metabolic regulator in the complex networks of reprogramming. PI3K signaling is a key in the mTOR upstream activation process, which has long been known as an essential mediator of trained immunity [31]. Akt/mTOR/HIF1α-dependent induction of aerobic glycolysis has been found to represent the metabolic basis of trained immunity [1]. In our study, enhanced mTOR signaling activation corresponded to increased expression levels of STING signaling pathway-related proteins after HKCA or DMXAA training. More importantly, inhibiting the mTOR signaling with rapamycin attenuated trained-PM functions, mainly characterized by decreased inflammatory responses after LPS stimulation and reduced phagocytosis and the bacterial killing ability upon *C. perfringens* challenge. Similarly, mTOR-dependent ROS production regulates the oxLDL-induced trained immunity in human monocytes [32]. A defect in HIF-1α recruitment of neonatal immune cells under low oxygen partial pressure leads to decreased phagocytosis and ROS production [33]. The application of rapamycin effectively inhibits the proinflammatory memory-like response of microglia induced by B cell-activating factor [34]. To further clarify the role of mTOR signaling in the process of STING-dependent trained immunity, we detected the expression of p-mTOR, p-AKT, and HIF1α. Compared with WT cells, STING^−/−^ PM exhibit decreased levels of the above proteins after HKCA training, approaching the levels seen in the non-training group. Hence, our investigation highlighted the significance of STING in promoting trained immunity induction for host defense against *C. perfringens* infection via the mTOR signaling pathway. Hopefully, these studies will provide candidate resources for the development of STING-based vaccine adjuvants, the design of dual memory vaccines which induce a strong persistent memory response, and preventive therapeutics against infectious disease.

### Supplementary Information

**Additional file 1: The secretion of TNF-α and IL-6 in WT and STING**^**-/-**^** PM after *C. perfringens* infection.** WT and STING^-/-^ PM were trained with HKCA and then were restimulated with *C. perfringens* at an MOI of 5 for the indicated times. **A**, **B** TNF-α and IL-6 production was measured in the supernatants of mouse PM. Data are shown as mean ± SEM. Data were pooled from 3 independent experiments. Statistical significance is indicated by *p < 0.05 and ***p < 0.001.

## Data Availability

All data generated or analyzed during this study are included in this published article.

## Abbreviations

*C. perfringens*: *Clostridium perfringens*
HKCA: heat-killed *Candida albicans*
STING: stimulator of interferon genes
WT: wild-type
PRR: pattern-recognition receptors
IFN: type I interferons
PM: peritoneal macrophages
PBS: phosphate-buffered saline
i.p.: intraperitoneally
CFU: colony-forming units
