# Correction: STING-dependent trained immunity contributes to host defense against *Clostridium perfringens* infection via mTOR signaling

**DOI:** 10.1186/s13567-024-01322-w

**Published:** 2024-06-13

**Authors:** Zhen-Zhen Liu, Cheng-Kai Zhou, Xiao-Qi Lin, Yu Gao, Xue-Yue Luo, Jia-Bao Zhang, Qi Yin, Liang Zhang, Jian-Gang Zhang, Xin An, Wei Chen, Yong-Jun Yang

**Affiliations:** State Key Laboratory for Diagnosis and Treatment of Severe Zoonotic Infectious Diseases, Key Laboratory for Zoonosis Research of the Ministry of Education, Institute of Zoonosis, and College of Veterinary Medicine, Jilin University, Changchun, 130062 China

**Correction: Veterinary Research (2024) 55:52** 10.1186/s13567-024-01301-1

Following publication of the original article [1], the authors identified an assembly error in the representative image of “WT-HKCA-PBS group” in Figure 7B.

The original article has been corrected.
