# Supplementary figures and images for: STING-dependent trained immunity contributes to host defense against Clostridium perfringens infection via mTOR signaling
Source: Vet Res. 2024 Apr 15;55:52. doi: 10.1186/s13567-024-01301-1 (PMC11017476; doi:10.1186/s13567-024-01301-1)

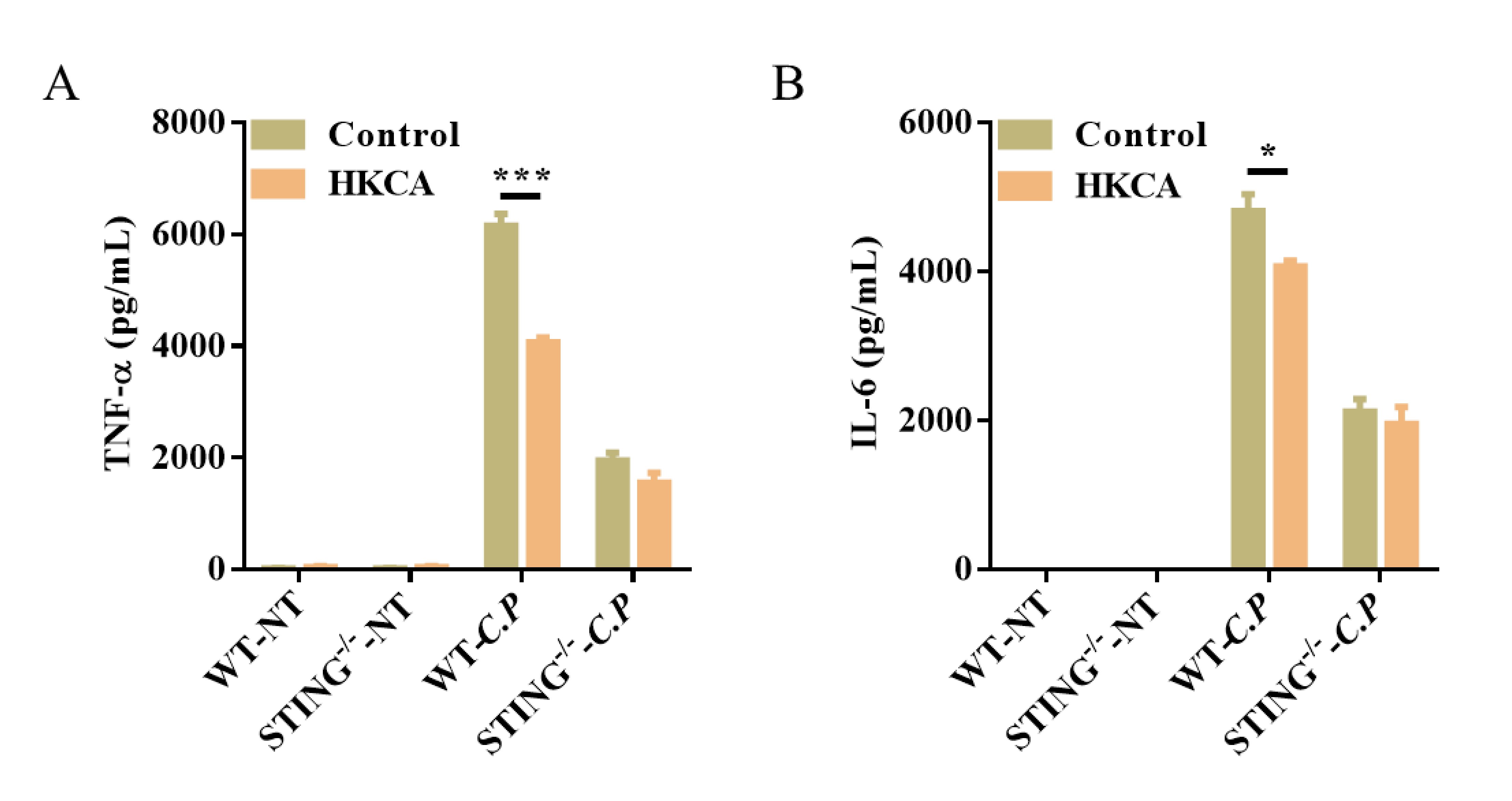

Supplement: Supplementary file 1 — Additional file 1: The secretion of TNF-α and IL-6 in WT and STING-/- PM after C. perfringens infection. WT and STING-/- PM were trained with HKCA and then were restimulated with C. perfringens at an MOI of 5 for the indicated times. A, B TNF-α and IL-6 production was measured in the supernatants of mouse PM. Data are shown as mean ± SEM. Data were pooled from 3 independent experiments. Statistical significance is indicated by *p < 0.05 and ***p < 0.001. [file 13567_2024_1301_MOESM1_ESM.tif]
